# Deep transcranial magnetic stimulation in combination with skin thermography in obesity: a window on sympathetic nervous system

**DOI:** 10.1007/s00592-022-01859-2

**Published:** 2022-02-17

**Authors:** Anna Ferrulli, Sara Gandini, Giulio Cammarata, Veronica Redaelli, Stefano Massarini, Concetta Macrì, Ileana Terruzzi, Daniele Cannavaro, Fabio Luzi, Livio Luzi

**Affiliations:** 1 Department of Endocrinology, Nutrition and Metabolic Diseases, IRCCS MultiMedica, Via Milanese, N. 300, 20099 Sesto San Giovanni (MI), Italy; 2 Department of Biomedical Sciences for Health, University of Milan, Milan, Italy; 3 Department of Experimental Oncology, European Institute of Oncology IRCCS, Milan, Italy; 4 Department of Biomedical, Surgical and Dental Sciences - One Health Unit, University of Milan, Milan, Italy

**Keywords:** Deep transcranial magnetic stimulation, Obesity, Temperature, Infrared thermography, Norepinephrine

## Abstract

**Aims:**

Obesity is known to be associated with an altered thermoregulation as well as a dysregulation of sympathetic nervous system (SNS). Considering the ability of deep transcranial magnetic stimulation (dTMS) to modulate the SNS, we hypothesized a potential role of dTMS in affecting thermoregulation in obesity. Aims of the study were to monitor the effect of a single session of dTMS on body temperature in subjects with obesity, and to correlate the dTMS-induced changes in body temperature with activation of the SNS (epinephrine and norepinephrine release).

**Methods:**

Twenty-nine subjects with obesity [5 M, 24 F; age 50 (IQR: 58, 38) yrs; BMI 36.1 (IQR: 33.9, 38.7) kg/m^2^] were randomized into 2 groups receiving a single session of high frequency stimulation (HF) or sham stimulation. Under neutral thermal conditions, infrared thermography was utilized to assess bilateral fingernail-beds and abdominal temperature.

**Results:**

During a single session HF, the average temperature of both fingernail-beds decreased. Right-hand temperature difference was statistically greater in HF *vs* Sham: median = – 1.45 (IQR: – 2.0, – 1.0)  °C for HF, *p = *0.009. While temperature variation in the fingernail-bed of left hand was not statistically significant in HF compared to Sham: median = – 1.26 (IQR: – 1.6, –0.5) °C, *p = *0.064. Concurrently, when estimating the effect of norepinephrine variation on temperature change of fingernail-bed of left hand, a borderline significant positive association was estimated (beta = 1.09, *p = *0.067) in HF.

**Conclusions:**

Deep TMS revealed to be effective in modulating temperature in subjects with obesity, partially reversing obesity-induced alterations in heat production and dissipation with a potential SNS-mediated mechanism.

## Introduction

Obesity is known to be associated with an altered thermoregulation [1, 2]. An increased resting metabolic caloric production [3, 4], combined with reduced heat dissipation due to the subcutaneous adipose tissue that acts as an insulating layer, has been shown in individuals with obesity [5]. Therefore, heat retention in areas of the body with greater adiposity is counteracted by an augmented heat release from the extremities, as the fingernail-beds of both hands to maintain euthermia in subject with obesity [5, 6].

Maintenance of a homeostatic body core temperature is a critical brain function accomplished by a complex neural network. The hypothalamus, specifically the preoptic anterior hypothalamus, represents the coordinating or central integration center for the thermoregulation [7]. It receives inputs from peripheral as well as from central thermoreceptors, which could be cold or warmth-responding [8]. A significant role in the thermoregulatory process has been played by the skin blood flow, which in turn is regulated by the autonomic nervous system (ANS) [9]. Specifically, two branches of the sympathetic nervous system (SNS) are mainly effectors of skin blood flow [10]: sympathetic vasoconstrictor nerves, which release norepinephrine (NE) and co-transmitters and are responsible for minor variations in skin blood flow occurring during most daily activities, and the sympathetic active vasodilator system that works via cholinergic nerve co-transmission, but in this case, the underlying mechanisms are incompletely understood [11].

Thermoregulatory arterio-venous shunt vasoconstriction is mainly mediated by local release of NE rather than alterations in systemic catecholamine concentrations [6]. Norepinephrine preferentially binds α1-adrenoceptors by inducing smooth muscle contraction and vasoconstriction. Similar responses occur with the NE binding to post-junctional α2-adrenoceptors located on some blood vessels. Conversely, a vasodilator effect has been observed when NE binds the post-junctional β2-adrenoceptors, although this effect of NE is relatively weak and counteracted by the more powerful α-adrenoceptor-mediated vasoconstriction [12]. Concerning epinephrine (EPI), a high affinity for smooth muscle β2-adrenoceptors, inducing vasodilation in some organs, has been shown; at higher concentrations, it can produce vasoconstriction by binding the α1- and α2-adrenoceptors [12].

The impact of ANS on thermoregulation could be mediated, not only by its regulatory effect on cutaneous blood flow, but also on brown adipose tissue (BAT) function. Specifically, the SNS regulates BAT function, mainly through the β1- and β3-adrenergic receptors involved in stimulating brown adipocyte proliferation and in activating mature brown adipocytes, respectively. ANS dysfunctions, specifically an increased sympathetic activity, have been demonstrated in individuals with obesity, favoring the development of complications in the cardiovascular system, as well as in the thermoregulation process [13, 14].

Although few data are available in humans, it is well known that also the opioid system plays an important role in regulating body temperature [15].

Noninvasive brain stimulation (NBS) has been introduced to alter human brain function in a safe, tolerable, and convenient way and has been employed in the treatment of various neuro-psychiatric disorders [16]. It includes repetitive transcranial magnetic stimulation (rTMS), transcranial direct current stimulation (tDCS), and a variant of TMS [i.e., deep transcranial magnetic stimulation (dTMS)], able to stimulate deeper brain regions as the insula. Recently, we demonstrated the safety and efficacy of dTMS, targeted to the prefrontal cortex (PFC) and insula bilaterally, in controlling food craving and reducing body weight, up to 1-year period in individuals with obesity [17, 18], through enhancing inhibitory capacity of PFC overeating behavior [19], and modulating intra-cerebral dopamine release. The potential for NBS to become an effective and safe strategy for the management of obesity has been confirmed by other randomized clinical trials [20].

Considering the ability of dTMS to modulate directly or indirectly (via cortical excitability) the ANS [21, 22], to promote neuro-hormonal peptides release, as the β-endorphin [23], and to potentially affect the leptin system by promoting weight loss (many hypothalamic neurons involved in regulating thermogenesis are also leptin sensitive) [24, 25], we hypothesized a potential role of dTMS in affecting thermoregulation in obesity, and in reversing obesity-induced alterations in body temperature. Presently, we propose the combination of dTMS with infrared thermography (IRT), as a new research tool for the detection of body temperature, with the following aims: 1. Monitoring the effect of a single session of high-frequency dTMS on body temperature in subjects with obesity, compared to a single session of sham stimulation; 2. correlating the dTMS-induced changes in body temperature with activation of the SNS (EPI and NE release).

To our knowledge, this is the first study that uses IRT to evaluate differences in skin temperature of different body areas in individuals with obesity, at rest condition and after a single session of dTMS.

## Materials and methods

### Study setting

This study was performed at the Endocrinology and Metabolic Diseases Division, IRCCS Policlinico San Donato, San Donato Milanese (MI), Italy, and is registered with ClinicalTrials.gov, number NCT03009695. The study was conducted in accordance with the ethical standards of the institutional research committee and with the 1964 Helsinki declaration and its later amendments. The study received approval by the local Institutional Review Board (Ethics Committee of San Raffaele Hospital, Milan, Italy). All participants provided written informed consent before participating in any study procedures.

Original study protocol was designed as a double-blind, sham-controlled, randomized clinical trial aimed at investigating the effects of a 5-week treatment with dTMS in reducing food craving and body weight in individuals with obesity, comparing high frequency (HF, 18 Hz) with low-frequency (LF, 1 Hz) stimulation and with Sham. The trial has been registered with ClinicalTrials.gov, number NCT03009695.

In 2019, we published preliminary results of the study, demonstrating the safety and efficacy of dTMS, along with a hypocaloric diet, in reducing body weight for up to 1 year in obese people [17]. In this study, statistical analysis highlighted poor efficacy of low-frequency stimulation in controlling food craving and reducing body weight in obesity. Therefore, after approval of a protocol amendment by the Ethics Committee, we discontinued recruitment to the LF group, and only enrolled in the HF and Sham groups.

### Study participants

Adult men and women (aged 22–65 years, inclusive), who referred to the Endocrinology and Metabolic Diseases outpatient clinic for overweight/obesity treatment from January 2017 until January 2020, were screened with a short interview to determine eligibility. Patient recruitment strategy involved direct interviews. Inclusion and exclusion criteria reported in Table 1.

**Table 1:** Inclusion and exclusion criteria of participants

Inclusion criteria	Exclusion criteria
Age 22–65 years	Personal or a family history of seizures
BMI 30–45 kg/m^2^	Organic brain disorders
	Psychiatric disorders according to DSM-5 criteria
	Implanted metal devices
	Abuse of substances other than nicotine
	Weight variation (> 3%) within three months prior the screening visit
	Current or recent (within 6 months prior the screening visit) treatment with anti-obesity medications or other medications for weight reduction
	Medications associated with lowered seizure threshold
	Type 1 diabetes or insulin-treated type 2 diabetes
	Fever and/or infectious state
	Pregnancy and breastfeeding
	Post-ovulatory phase for women of childbearing age

BMI body mass index, *DSM* diagnostic and statistical manual of mental disorders

### Randomization and masking

Patients fulfilling all inclusion/exclusion criteria were randomized to one of two experimental groups: HF or Sham. Allocation in the two groups was performed according to a randomization sequence generated by a computerized program. The randomization code was only given to the treating investigator at the first treatment session by an independent investigator not involved with any other aspect of the trial. Participants and other investigators were unaware of the type of treatment assignment. Magnetic cards encoding for real or sham stimulation were used to activate the dTMS device or not, according to the randomization sequence. Both real and sham stimulation produced identical sounds and scalp sensations during the sessions.

### Study design

This study was designed as a double-blind, sham-controlled, randomized protocol aimed to investigate the acute effects of a single HF dTMS session on body temperature, measured by IRT, and to identify potential correlations between temperature variations and serum level changes of EPI, NE, β-endorphin, in individuals with obesity.

### Repetitive deep transcranial stimulation procedure (dTMS)

The repetitive dTMS was performed by a trained physician using a Magstim Rapid^2^TMS (The Magstim Co. Ltd., Whitland, Carmarthenshire, the UK) stimulator equipped with an H-shaped coil, specifically designed to bilaterally stimulate the PFC and the insula [26, 27]. Magnetic cards encoding for real or sham stimulation were used to activate the dTMS device. Both real and sham stimulation produced identical sounds and scalp sensations during the sessions.

The characteristics of the stimulation protocols are the same as those used in the study by Ferrulli et al. [17]. For active stimulation, sessions consisted of 80 trains of 18 Hz, each lasting 2 s, with an intertrain interval of 20 s. The HF treatment duration was 29.3 min with 2880 pulses in total. Sham stimulation entailed the same coil placement and procedures as the active condition; however, the device automatically turned off after 15 s of active stimulation, producing similar acoustic artifacts and scalp sensations.

### Infrared thermography

Thermographic images of fingernail-beds of both hands and of abdominal skin were acquired by an AVIO R500EXPro Thermal Camera. The sensitivity of the camera was < 0.025 °C, and the images had dimensions of 640 × 480 pixels. The IRT technique is based on the principle that the amount of energy radiated depends on the surface temperature of the object and the emissivity of the object’s surface [33]. The camera detects the infrared energy from an object and uses this information to estimate its temperature. Last decades witnessed a steady increase in the clinical application of IRT technique to obtain correlations between the thermal physiology and skin temperature [28]. IRT has been successfully used in diagnosis of breast [29, 30] or skin [31] cancer, diabetes neuropathy [32] and peripheral vascular disorders [33].

We decided to measure the temperature at the fingernail-beds of the hands and abdomen as they represent the areas where the temperature varies more significantly in individuals with obesity compared to healthy controls, in agreement with previous studies [5, 34]. The plane of the infrared camera’s lens was positioned parallel to the plane of the body region to detect, at 40–60 cm. Participants were asked to remain as still as possible during the periods of infrared imaging, to reduce motion artifacts.

Moreover, on the day of the examination, the participants were asked to not apply any type of skin cream or alcohol-based products, to not practice physical activity, to not ingest food or alcohol, to not smoke, and to not exposure to UVA. The testing room was comfortable and acclimated so that the participant felt calm before undergoing the test, in order to avoid physiological changes (sweat or tachycardia, dizziness, etc.) and reach a thermal equilibrium. The participants were asked to remain seated for at least twenty minutes before the examination, avoiding, during the wait, inappropriate postures like crossed legs or arms.

In our study, the average temperature of the test room was maintained between 21 °C and 23 °C, and there was no heat source close to the subject. Doors and windows were closed during the tests to avoid uncontrolled airflow in the room. In addition, there were not objects that generated any thermal interference.

### Laboratory measurements

After placing a plastic catheter into the forearm vein, blood specimen was drawn for the measurement of EPI (pg/mL), NE (ng/mL) and β-endorphin (ng/mL) before (T0) and after a single dTMS session (T1). Enzyme-linked immunosorbent assay (ELISA) kits were used to assess EPI and NE (Elabscience Biotechnology Co. Ltd, Wuhan, China); β-endorphins levels were measured using commercially available enzyme immunoassay (EIA) kits (Phoenix Pharmaceuticals, Burlingame, CA, the USA).

### Statistical analysis

Data for each parameter were expressed as median and interquartile range (IQR). Comparisons of patients’ characteristics between treatment arms at baseline were evaluated with Wilcoxon signed-rank test.

Changes in time (t_1_-t_0_) in temperatures measurements were compared by treatments arms. The associations of changes in time in temperatures measurements with neurotransmitters changes were also investigated looking at the effects of treatment arms.

For left and right hand, linear regression models were used to determine associations between neurotransmitters changes and temperature changes and in time. We also investigated the role of confounders such as sex, body mass index (BMI) and age. Residuals were checked to investigate normal distribution of fully adjusted models. For all tests, differences were considered statistically significant at *p* ≤ 0.05. All statistical analyses were conducted using R (version 4.1.0) software.

## Results

### Subjects

A total of twenty-nine patients with obesity met the criteria and were enrolled in the study protocol. In particular: 24 F, 5 M with median age 50 (IQR: 58, 38) yrs, median body weight 97.6 (IQR: 87.9, 104.1) kg, median BMI 36.1 (IQR: 33.9, 38.7) kg/m^2^. Out of the 29 patients, 27 were right-handed, and only 2 patients were left-handed. Seventeen patients were enrolled in HF arm. In particular: 14 F, 3 M with median age 48 (IQR: 38.0, 55.0) yrs, median body weight 98 (IQR: 87.7, 103.9) kg, BMI 35.4 (IQR: 33.8, 36.6) kg/m^2^. Twelve patients were enrolled in Sham arm. In particular: 10 F, 2 M with median age 55.0 (IQR: 39.7, 58.5) yrs, median body weight 97.1 (IQR: 94.7, 104.7) kg, median BMI 38.2 (IQR: 35.1, 38.9) kg/m^2^. At baseline, no significant differences in gender, age, body weight and BMI were found between the two arms (Table 2).

**Table 2:** Anthropometric measures of the total population of subjects with obesity enrolled in the study

	Total (29)	HF (17)	SHAM (12)	*p* value
Patients, n (%)	100%	58.6%	41.4%	
*Gender*				
Female, n (%)	24 (82.8%)	14 (82.4%)	10 (83.4%)	1
Male, n (%)	5 (17.2%)	3 (17.6%)	2 (16.6%)	
*Age (yrs)*				
Median (IQR*)*	50.0 (38.0, 58.0)	48.0 (38.0, 55.0)	55.0 (39.7, 58.5)	0.49
*Body weight (kg)*				
Median (IQR)	97.6 (87.9, 104.1)	98.0 (87.7, 103.9)	97.1 (94.7, 104.7)	0.89
*BMI (kg/m*^*2*^*)*				
Median (IQR)	36.1 (33.9, 38.7)	35.4 (33.8, 36.6)	38.2 (35.0, 38.9)	0.18

Signif. Codes: *p* ≤ 0.001***, *p* ≤ 0.01**, *p* ≤ 0.05*

Data are expressed as median (IQR). Comparisons between the 2 arms of treatment (HF, Sham) have been performed by χ^2^ test (Gender) and by Wilcoxon signed-rank test (Age, Body weight, BMI), and their p values are reported

*HF* high Frequency*,* BMI body mass index

Under neutral thermal conditions, fingernail-bed temperature of both hands and abdominal skin temperature, and neuropeptides (EPI, NE and β-endorphin) was evaluated acutely before (T0) and after (T1) a single HF or sham dTMS session.

### Body temperature variations

All values are reported as median change (t_1_-t_0_) and IQR of changes (t_1_-t_0_). During a single session of HF dTMS, the fingernail-bed of both hands’ temperature (°C) decreased in both arms. Right-hand temperature difference (t_1_-t_0_) was statistically greater in HF vs Sham: median = -1.45 (IQR: -2.0, – 1.0) °C for HF, *p = *0.009. Left-hand temperature time changes (t_1_-t_0_) were also greater in the HF arm: median = – 1.26 (IQR: – 1.6, – 0.5) °C, but the difference between arms was not statistically significant *p = *0.064 (Table 3).

**Table 3:** Temperature and neuropeptides measures of each arm and their changes over time (t1-t0)

	HF T_0_	HF T_1_	SHAM T_0_	SHAM T_1_	Δ HF	Δ SHAM	*p* value
*Fingernail-bed of right-hand temperature*							
Median (IQR)	34.5 (33.4, 34.9)	33.0 (31.5, 33.8)	33.8 (31.0, 34.3)	33.0 (30.1, 33.8)	-1.45 (-2.0, -1.0)	-0.5 (-1.0, -0.0)	0.009**
*Fingernail-bed of left-hand temperature*							
Median *(IQR)*	34.2 (33.4, 34.8)	33.1 (31.2, 33.7)	34.0 (31.2, 34.6)	33.3 (31.7, 34.0)	-1.26 (-1.6, -0.5)	-0.4 (-0.8, -0.1)	0.064
*Abdominal skin temperature*							
Median (IQR)	34.1 (33.4, 34.7)	34.6 (33.2, 35.3)	33.5 (33.3, 33.7)	34.1 (33.2, 34.2)	0.2 (-0.5, 1.0)	0.3 (-0.3, 0.5)	0.869
*Norepinephrine*							
Median (IQR)	3.64 (2.63, 4.89)	3.27 (1.51, 4.73)	2.06 (1.38, 4.48)	3.03 (1.51, 4.73)	0.09 (-0.12, 0.25)	-0.01 (-0.06, 0.09)	0.804
*Β-Endorphin*							
Median (IQR)	0.46 (0.450, 0.54)	0.51 (0.48, 0.54)	0.52 (0.48, 0.59)	0.52 (0.47, 0.56)	0.01 (-0.01, 0.05)	-0.00 (-0.06, 0.03)	0.328
*Epinephrine*							
Median (IQR)	327.35 (121.7, 636.5)	283.02 (115.9, 635.6)	267.26 (111.9,456.0)	366.80 (222.3, 667.2)	-2.84 (-34.61, 7.70)	11.75 (-26.72, 31.63)	0.238

Signif. Codes: p ≤ 0.001***, p ≤ 0.01**, p ≤ 0.05*

Data are expressed as median (Q1-Q3). Comparisons between the 2 arms of treatment (HF, Sham) have been performed by Wilcoxon signed-rank test. P value evaluates the differences between treatment arms in norepinephrine, endorphin and epinephrine changes over time (t_1_-t_0_)

Temperature measures (°C) and epinephrine (pg/mL), norepinephrine (ng/mL), β-endorphin measures (ng/mL) in each arm and their changes over time (t_1_-t_0_)

**Fig. 1:**
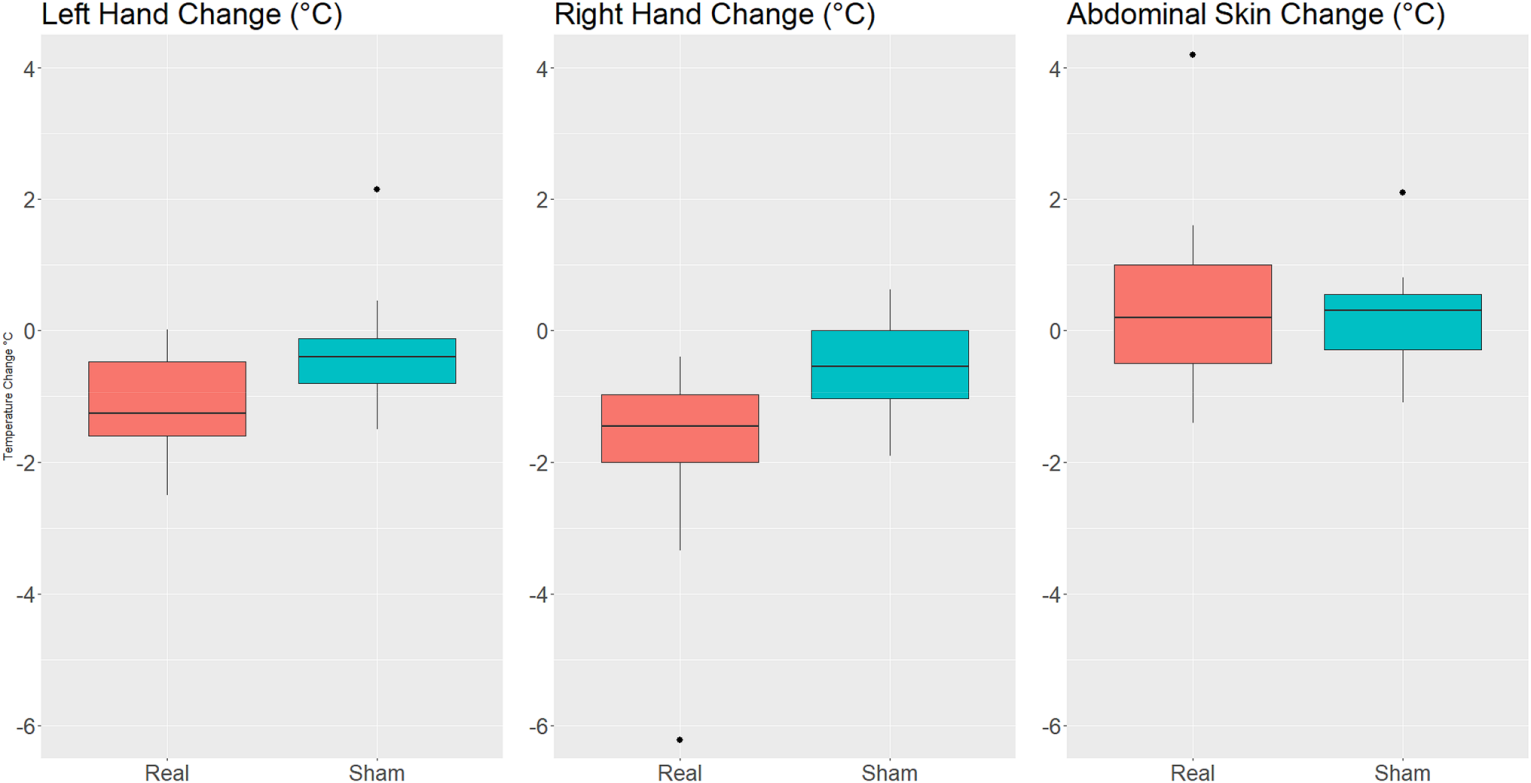
Temperature change (t1-t0) boxplot: left hand, right hand, abdominal skin. After a single HF dTMS session, the difference in the fingernail-bed temperature of left hand (t1-t0) was greater in the HF arm: median = -1.26 (IQR: -1.6, -0.5) °C and the differences between arms were borderline statistically significant *p = *0.064. Right-hand temperature difference (t1-t0) was statistically greater in HF vs Sham: median = -1.45 (IQR: -2.0, -1.0) °C for HF, *p = *0.009. A non-significant increase in abdominal skin temperature was observed in HF arm comparing to Sham arm. For HF arm: 0.2 (IQR: -0.5, 1.0) °C vs Sham arm 0.3 (IQR: -0.3, 0.5) °C, *p = *0.869

**Fig. 2:**
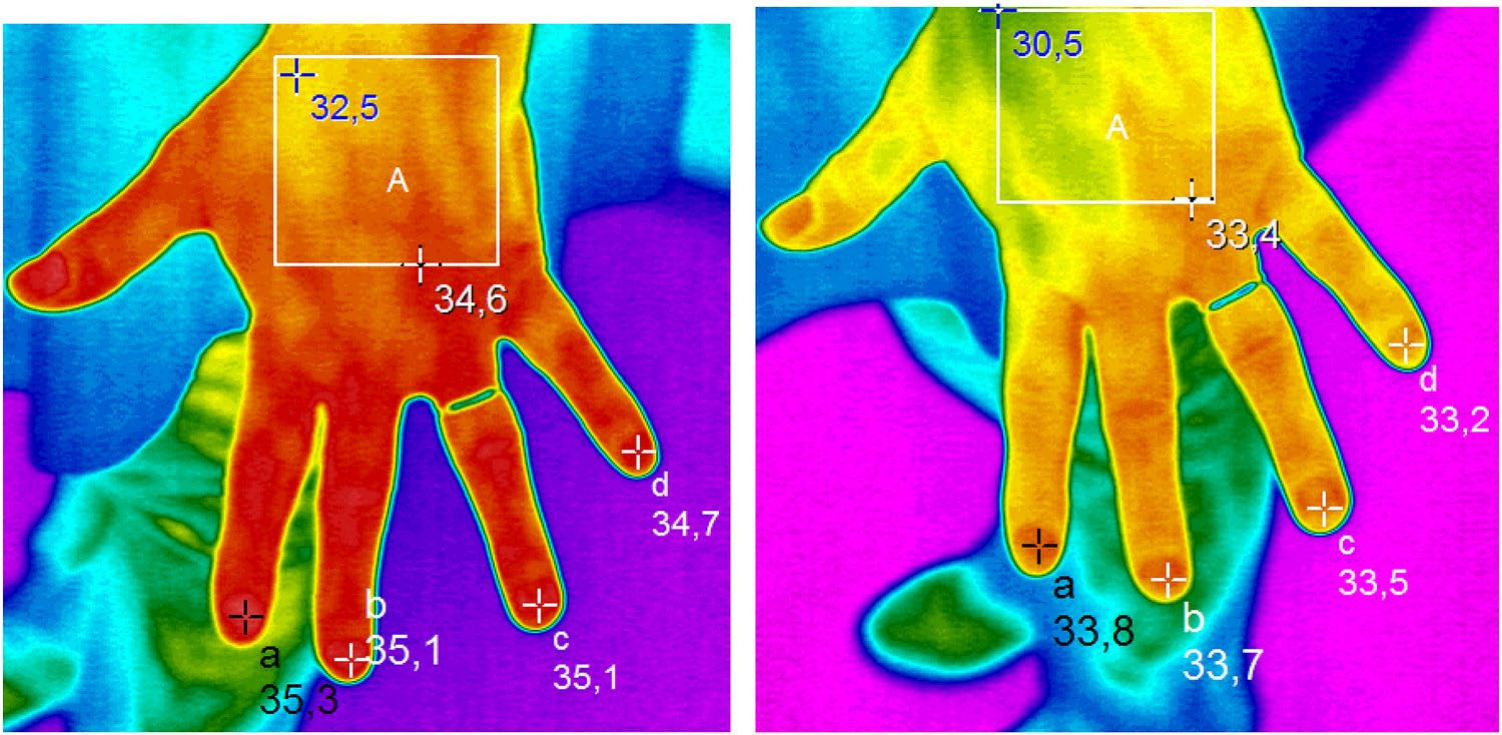
Fingernail-beds of left-hand temperature after a single HF dTMS session detected by infrared thermography

During a single treatment session, a non-significant increase in abdominal skin temperature was observed in HF arm comparing to Sham arm. For HF arm: 0.2 (IQR: –0.5, 1.0) °C vs Sham arm 0.3 (IQR: – 0.3, 0.5) °C, *p = *0.869 (Table 3). Boxplots of the difference in left-hand temperature, right-hand temperature and abdominal skin temperature are displayed in Fig. 1. IFR images of fingernail-bed of right hand before and after a single dTMS session have been also reported (Fig. 2).

### EPI, β-endorphin and NE variations

After a single HF dTMS session, the median value of NE change (t_1_-t_0_) was 0.09 (IQR: -0.12, 0.25) ng/mL. The median value of endorphin change (t_1_-t_0_) was 0.01 (IQR: – 0.01, 0.05) ng/mL. The median value of EPI change (t_1_-t_0_) was – 2.84 (IQR: – 34.61, 7.70) pg/mL. No significant differences between the HF and Sham arms were found (Table 3).

## Regression models results on neurotransmitters changes

To understand if, in the sample, a change (t_1_-t_0_) in neurotransmitters affects temperature (t_1_-t_0_), linear models for left-hand and right-hand temperature change were estimated. For NE, temperature changes (t_1_-t_0_) in left hand and right hand are reported in Tables 4 and 5, respectively. For β-endorphin, temperature changes (t_1_-t_0_) in left hand and right hand are reported in Tables 6 and 7, respectively. Finally, for EPI, temperature changes in left hand and right hand are reported in Tables 8 and 9, respectively.

**Table 4:** Left hand: linear regression models for the effect of norepinephrine change (t_1_-t_0_) on temperature change (t_1_-t_0_)

Left Hand Temp. (Δ°C)	Model 1	*p* value	Model 2 (HF)	*p* value	Model 3 (Sham)	*p* value
Predictors	Beta (CI 95%)		Estimates (CI 95%)		Estimates (CI 95%)	
Intercept	– 1.30 (– 1.69,– 0.92)	< 0.001***	– 1.37 (– 1.77, – 0.98)	< 0.001***	– 0.50 (– 1.06, 0.06)	0.071
Norepinephrine (Δ)	0.30 (– 0.28, 0.87)	0.296	1.09 (– 0.09, 2.28)	0.067	0.05 (– 0.68, 0.78)	0.878
Sham (vs HF)	0.75 (0.13, 1.37)	0.020*				
Observations (n)	21		13		8	

Signif. codes: *p* ≤ 0.001***, *p* ≤ 0.01**, *p* ≤ 0.05*

Model 1: Whole cohort linear regression model of norepinephrine change (t_1_-t_0_) on left hand temperature change (t_1_-t_0_) by ARM

Model 2 (HF): Whole cohort linear regression model of norepinephrine change (t_1_-t_0_) on left hand temperature change (t_1_-t_0_) for HF arm

Model 3 (Sham): Whole cohort linear regression model of norepinephrine change (t_1_-t_0_) on left hand temperature change (t_1_-t_0_) for Sham arm

**Table 5:** Right hand: linear regression models for the effect of norepinephrine change (t_1_-t_0_) on temperature change (t_1_-t_0_)

Right Hand Temp. (Δ°C)	Model 1	*p *value	Model 2 (HF)	*p *value	Model 3 (Sham)	*p *value
Predictors	Beta (CI 95%)		Estimates (CI 95%)		Estimates (CI 95%)	
Intercept	– 1.73 (– 2.49,– 0.97)	< 0.001***	– 1.78 ( – 2.75, – 0.81)	0.002*	– 0.67 ( – 1.36, 0.02)	0.055
Norepinephrine (Δ)	0.14 (– 1.00, 1.28)	0.799	0.72 ( – 2.21, 3.64)	0.601	– 0.04 ( – 0.94, 0.87)	0.921
Sham (vs HF)	1.03(– 0.20, 2.25)	0.096				
Observations (n)	21		13		8	

Signif. codes: p ≤ 0.001***, p ≤ 0.01**, p ≤ 0.05*

Model 1: Whole cohort linear regression model of norepinephrine change (t_1_-t_0_) on right hand temperature change (t_1_-t_0_) by ARM

Model 2 (HF): Whole cohort linear regression model of norepinephrine change (t_1_-t_0_) on right hand temperature change (t_1_-t_0_) for HF arm

Model 3 (Sham): Whole cohort linear regression model of norepinephrine change (t_1_-t_0_) on right hand temperature change (t_1_-t_0_) for Sham arm

**Table 6:** Left Hand: Linear regression models for endorphin change (t_1_-t_0_) effect on temperature change (t_1_-t_0_)

Left Hand Temp. (Δ°C)	Model 1	*p* value	Model 2 (HF)	*p* value	Model 3 (Sham)	*p* value
Predictors	Beta (CI 95%)		Beta (CI 95%)		Beta (CI 95%)	
Intercept	– 1.27 (– 1.67, – 0.87)	< 0.001***	– 1.29 (– 1.74, – 0.83)	< 0.001***	– 0.54 (– 1.07, – 0.01)	0.046*
Endorphin (Δ)	– 0.49 (– 4.68, 3.71)	0.811	0.40 (– 5.15, 5.96)	0.876	– 2.93 (– 11.18, 5.32)	0.418
Sham (vs HF)	0.77 (0.12, 1.42)	0.023*				
Observations (n)	21		13		8	

Signif. codes: *p* ≤ 0.001***, *p* ≤ 0.01**, *p* ≤ 0.05*

Model 1: Whole cohort linear regression model of endorphin change (t_1_-t_0_) on left hand temperature change (t_1_-t_0_) by ARM

Model 2 (HF): Linear regression model of endorphin change (t_1_-t_0_) on left hand temperature change (t_1_-t_0_) for subjects in HF arm

Model 3 (Sham): Linear regression model of endorphin change (t_1_-t_0_) on left hand temperature change (t_1_-t_0_) for subjects in Sham arm

**Table 7:** Right Hand: Linear regression models for endorphin change (t_1_-t_0_) on temperature change (t_1_-t_0_)

Right Hand Temp. (Δ°C)	Model 1	*p* value	Model 2 (HF)	*p* value	Model 3 (Sham)	*p* value
Predictors	Beta (CI 95%)		Beta (CI 95%)		Beta (CI 95%)	
Intercept	– 1.77 (– 2.53, – 1.01)	< 0.001***	– 1.80 (– 2.74, – 0.85)	0.002**	– 0.70 (– 1.39, – 0.02)	0.045*
Endorphin (Δ)	2.72 (– 5.24, 10.68)	0.482	4.30 (– 7.23, 15.83)	0.429	– 1.62 (– 12.28, 9.05)	0.723
Sham (vs HF)	1.14 (– 0.10, 2.38)	0.068				
Observations (n)	21		13		8	

Signif. codes: p ≤ 0.001***, *p* ≤ 0.01**, *p* ≤ 0.05*

Model 1: Whole cohort linear regression model of endorphin change (t_1_-t_0_) on right hand temperature change (t_1_-t_0_) by ARM

Model 2 (HF): Linear regression model of endorphin change (t_1_-t_0_) on right hand temperature change (t_1_-t_0_) for subjects in HF arm

Model 3 (Sham): Linear regression model of endorphin change (t_1_-t_0_) on right hand temperature change (t_1_-t_0_) for subjects in Sham arm

**Table 8:** Left Hand: Linear regression models for epinephrine change (t_1_-t_0_) effect on temperature change (t_1_-t_0_)

Left Hand Temp. (Δ°C)	Model 1	*p* value	Model 2 (HF)	*p* value	Model 3 (Sham)	*p* value
Predictors	Beta (CI 95%)		Beta (CI 95%)		Beta (CI 95%)	
Intercept	– 1.37 (– 1.75, – 0.98)	< 0.001***	– 1.36 (– 1.82, – 0.90)	< 0.001***	– 0.41 (– 0.94, 0.12)	0.106
Epinephrine (Δ)	– 0.0009 (– 0.003, 0.001)	0.386	0.0002 (– 0.010, 0.010)	0.966	– 0.0009 (– 0.003, 0.001)	0.347
Sham (vs HF)	0.95 (0.31, 1.59)	0.006**				
Observations (n)	20		12		8	

Signif. codes: *p* ≤ 0.001***, *p* ≤ 0.01**, *p* ≤ 0.05*

Model 1: Whole cohort linear regression model of epinephrine change (t_1_-t_0_) on left hand temperature change (t_1_-t_0_) by ARM

Model 2 (HF): Linear regression model of epinephrine change (t_1_-t_0_) on left hand temperature change (t_1_-t_0_) for subjects in HF arm

Model 3 (Sham): Linear regression model of epinephrine change (t_1_-t_0_) on left hand temperature change (t_1_-t_0_) for subjects in Sham arm

**Table 9:** Right Hand: Linear regression models for epinephrine change (t_1_-t_0_) effect on temperature change (t_1_-t_0_)

Right Hand Temp (Δ°C)	Model 1	*p* value	Model 2 (HF)	*p* value	Model 3 (Sham)	*p* value
Predictors	Beta (CI 95%)		Beta (CI 95%)		Beta (CI 95%)	
Intercept	– 1.83 (– 2.62, – 1.04)	< 0.001***	– 1.77(– 2.78, – 0.75)	0.003**	– 0.64 (– 1.34, 0.06)	0.066
Epinephrine (Δ)	0.0004 (– 0.004, 0.004)	0.988	0.007(– 0.016, 0.030)	0.538	– 0.0004 (– 0.003, 0.003)	0.757
Sham (vs HF)	1.16 (– 0.14, 2.46)	0.078				
Observations (n)	20		12		8	

Signif. codes: *p* ≤ 0.001***, *p* ≤ 0.01**, *p* ≤ 0.05*

Model 1: Whole cohort linear regression model of epinephrine change (t_1_-t_0_) on right hand temperature change (t_1_-t_0_) by ARM

Model 2 (HF): Linear regression model of epinephrine change (t_1_-t_0_) on right hand temperature change (t_1_-t_0_) for subjects in HF arm

Model 3 (Sham): Linear regression model of epinephrine change (t_1_-t_0_) on right hand temperature change (t_1_-t_0_) for subjects in Sham arm

For each neurotransmitter, for each hand, three models were estimated. Model 1 presents estimates considering as dependent variable temperature change (t_1_-t_0_) and as independent variables each neurotransmitter change (t_1_-t_0_) and trial ARM. Model 2 and 3 present the results by treatment arm, HF and Sham, respectively.

### Norepinephrine

The effect of NE change (t_1_-t_0_) on left hand temperature change on (t_1_-t_0_) is reported in Table 4.

**Fig. 3:**
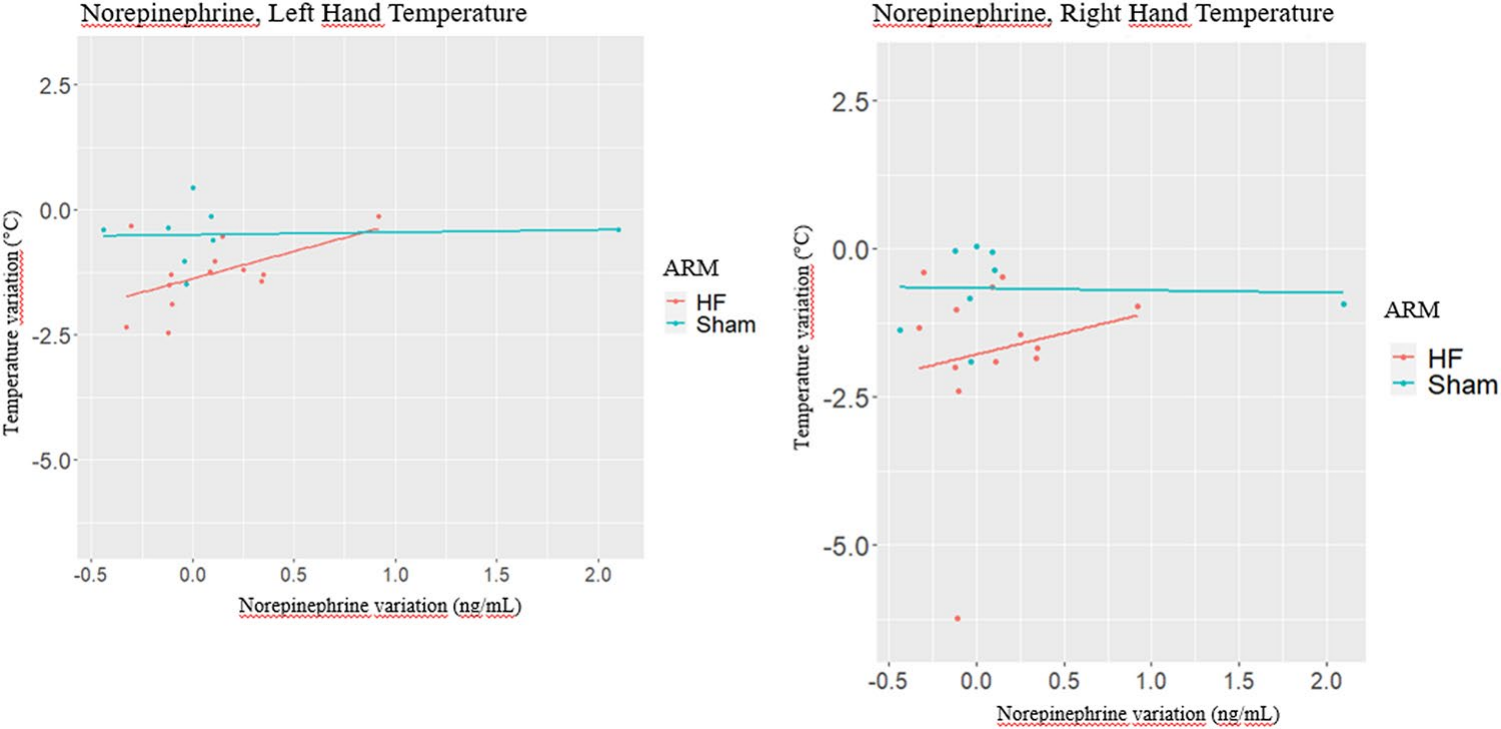
Effects of norepinephrine changes on temperature changes of fingernail-beds of both left and right hand in HF group. Linear regression model (Model 2) estimating the effect of NE’s change (t1-t0) on temperature change of fingernail-beds of left hand (t1-t0) for the HF treated arm showed a borderline significant positive association (beta = 1.09, *p = *0.067) (Fig. 3A). About the fingernail-bed temperature of right hand, in Model 2, the effect for HF group was positive (beta = 0.72, *p = *0.601), while it became negative for the Sham group (beta = -0.04, *p = *0.921), although it was not statistically significant. Figure 3B displays the different regression lines estimated for HF and Sham arms in the right hand

Model 1 reports a non-significant (beta = 0.30, *p = *0.296) effect of NE change (t_1_-t_0_) on temperature change (t_1_-t_0_) as well as a significant positive effect for treatment arm (beta = 0.75, *p = *0.020). To have an insight if a different effect for NE change exists by trial arm, we presented the stratified analysis by treatment arms in Model 2 and 3. In Model 2, when estimating the effect of NE change (t_1_-t_0_) on temperature change (t_1_-t_0_) only for the HF treated arm, a borderline significant positive association was estimated (beta = 1.09, *p = *0.067). In Model 3, the effect on temperature change (t_1_-t_0_) of NE change (t_1_-t_0_) for Sham arm was lower than the one estimated in the HF (beta = 0.05, *p = *0.878). The different effects of NE change (t_1_-t_0_) on temperature change (t_1_-t_0_) in left hand for different arms are displayed in Fig. 3A.

Estimates for right hand are reported in Table 5. In Model 1, a non-significant association was found for NE change (t_1_-t_0_) (beta = 0.14, *p = *0.799). The treatment effect was estimated as no significant as well (beta = 1.03, *p = *0.096). To see if, like left hand, a different effect of NE change (t_1_-t_0_) on temperature change (t_1_-t_0_) was estimated, Model 2 and Model 3 report the estimates by trial arm. In Model 2, the effect for HF group is positive (beta = 0.72, *p = *0.601), while it becomes negative for the Sham group (beta = -0.04, *p = *0.921). Figure 3B displays the different regression lines estimated for HF and Sham arms (Table 5).

### Β-Endorphin

Temperature changes (t_1_-t_0_) in left hand and right hand are also investigated as functions of β-endorphin changes (t_1_-t_0_) (Tables 6, 7, respectively).

For left hand, no effect of β-endorphin change (t_1_-t_0_) on temperature change (t_1_-t_0_) was estimated (beta = -0.49, *p = *0.811). A significant treatment effect is estimated (beta = 0.77, *p = *0.002). Given this result, to understand if β-endorphin change (t_1_-t_0_) affects temperature change (t_1_-t_0_) differently in HF and Sham arm, Model 2 and Model 3 estimate the effect by trial arm. For HF arm, the effect of β-endorphin change (t_1_-t_0_) on temperature change (t_1_-t_0_) is positive (beta = 0.40, *p = *0.876), while it becomes negative for Sham arm (-2.93, *p = *0.418) (Table 6).

**Fig. 4:**
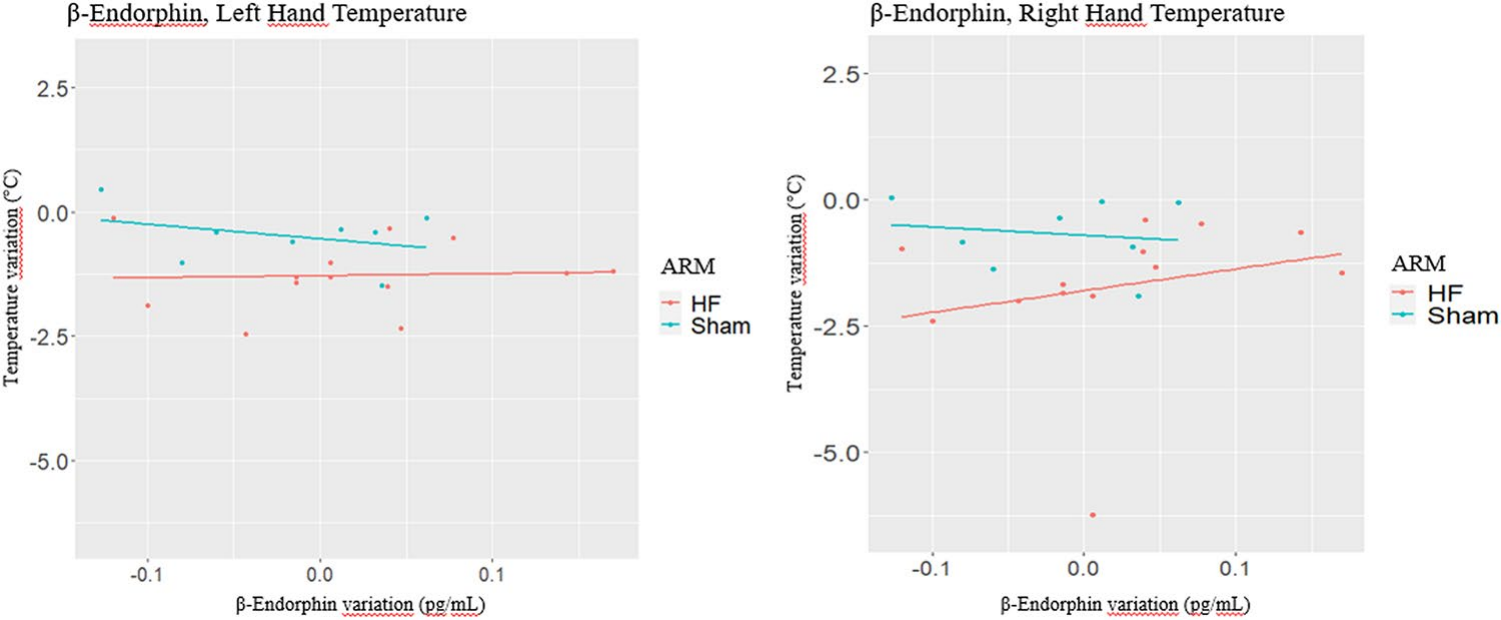
Effects of β-endorphin changes on temperature changes of fingernail-beds of both left- and right hand in HF group. For the left hand, the effect of β-endorphin change (t1-t0) on temperature change (t1-t0) was positive (beta = 0.40, *p = *0.876) for HF, while it became negative for Sham arm (-2.93, *p = *0.418) (Fig. 4 A). About the fingernail-bed temperature of right hand, in Model 2, the effect for HF group was positive (beta = 4.30, *p = *0.429), while in Model 3, it is estimated as negative for Sham arm (beta = -1.62, *p = *0.723). Figure 4B displays the opposite effects reported in Model 2 and Model 3

In Fig. 4A, the different effects of β-endorphin changes (t_1_-t_0_) on temperature change (t_1_-t_0_) for each trial arm in left hand are displayed.

Similarly, for right hand, no effect of β-endorphin change (t_1_-t_0_) on temperature change (t_1_-t_0_) was found (beta = 2.72, *p = *0.482) and a borderline significant treatment effect was estimated (beta = 1.14, *p = *0.068). As for left hand, when stratified by trial arm, an opposite effect for each arm is estimated. In fact, in Model 2, the effect of β-endorphin change (t_1_-t_0_) on temperature change (t_1_-t_0_) in HF arm is positive (beta = 4.30, *p = *0.429), while in Model 3, it is estimated as negative for Sham arm (beta = -1.62, *p = *0.723). Figure 4B displays the opposite effects reported in Model 2 and Model 3 (Table 7).

### Epinephrine

**Fig. 5:**
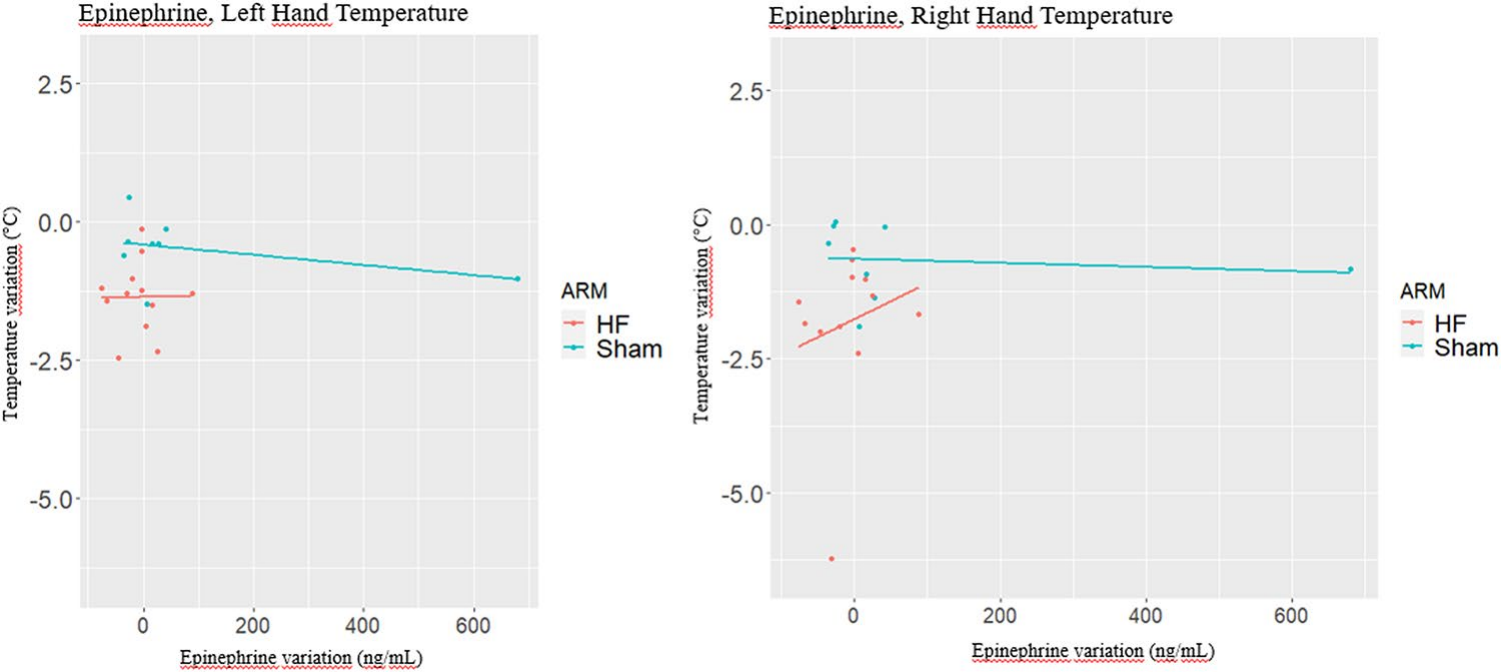
Effects of epinephrine changes on temperature changes of fingernail-beds of both left- and right hand in HF group. For the left hand, the effect of epinephrine change (t1-t0) on temperature change (t1-t0) was positive (beta = 0.0002, *p = *0.966) for HF arm, while it became negative for Sham arm (beta = -0.0009, *p = *0.347). Figure 5A displays the different effects estimated for HF and Sham arms (Table 8). About the fingernail-bed temperature of right hand, in Model 2, the effect for HF group was positive (beta = 0.007, *p = *0.538), while it became negative in Sham arm (beta = 0.0004, *p = *0.757). Figure 5B reports the different effects by trial arm

The effect of EPI change (t_1_-t_0_) on right hand and left-hand temperature change (t_1_-t_0_) is reported in Table 8 and 9, respectively. For left hand, Model 1 estimates no effect for EPI change (t_1_-t_0_) on temperature change (t_1_-t_0_) (beta = 0.00, *p = *0.386). A significant treatment effect is estimated (beta = 0.95, *p = *0.006). Therefore, to understand if a different effect by trial arm might be present in the sample, Models 2 and 3 present an analysis by treatment arm. In Model 2, a positive effect of EPI change (t_1_-t_0_) on temperature change (t_1_-t_0_) is estimated (beta = 0.0002, *p = *0.966) for HF arm, while in Model 3, a negative of EPI change (t_1_-t_0_) on temperature change (t_1_-t_0_) effect is estimated (beta = -0.0009, *p = *0.347). Figure 5A displays the different effects estimated for HF and Sham arms (Table 8).

Similarly, for right hand, no effect for EPI change (t_1_-t_0_) on temperature change (t_1_-t_0_) was estimated (beta = 0.0004, *p = *0.988) and a borderline significant treatment effect was estimated (beta = 1.16, *p = *0.078) in Model 1. As done for left hand, to understand whether a different effect of EPI change (t_1_-t_0_) on temperature change (t_1_-t_0_) is estimated by trial arm, Model 2 reports the effect for HF arm and Model 3 reports the effect for Sham arm. In HF arm, the effect is estimated as positive (beta = 0.007, *p = *0.538), while it becomes negative in Sham arm (beta = 0.0004, *p = *0.757). Figure 5B reports the different effects by trial arm.

Age, BMI, and sex were also found to be not statistically significantly associated with any temperature change.

## Discussion

In the present study, we examined the effects on body temperature (fingernail-bed of both hands and abdominal skin) of a single treatment session with dTMS over the PFC and the insula, bilaterally, using either HF or sham stimulation in individuals with obesity. Secondarily, we investigated possible correlations between the dTMS-induced variations in body temperature and the EPI, NE and β-endorphin level changes, the last two being suggestive of SNS activation. The novelty of this study consists in the combination of dTMS with IRT as a system to correlate SNS activation (EPI and NE blood levels) with the decrease in skin temperature of selected regions.

First achievement of our study is the demonstration that a single session of HF dTMS is effective in acutely modulating body temperature by decreasing the fingernail-bed temperature of right hand but not of the left hand (in which only a downward trend was observed) in individuals with obesity, thus partially reversing obesity-induced alterations in heat production and dissipation. No acute dTMS-induced effect has been observed on abdominal temperature. Obesity is characterized by a general down-regulation of heat turnover, with an impaired heat production and dissipation [5]. The impairment of heat homeostasis is directly proportional to the degree of obesity [35, 36]. The mechanisms underlying the alteration of heat turnover regulation are several. Heat production takes place mainly in skeletal muscles mass [37]. In the natural history of obesity, muscle mass can be either increased (young subjects) or decreased in absolute terms when sarcopenia develops in older individuals with obesity [38]. To note that muscle mass is always reduced in relative terms, namely related to surface area or total body weight in subjects with obesity [39]. This leads to a reduction of basal metabolic rate and heat production. Concerning heat dissipation, this takes place mainly at the body extremities, site with less fat deposition [5]. Although our evaluation was performed acutely, before and after a single dTMS session, these findings lead to hypothesize that dTMS may play a modulatory action on body temperature.

Despite a decreasing trend occurred also in the temperature fingernail-bed of the left hand, the temperature variation turned out to be significant only in the right hand. Some hypotheses to explain this difference can be formulated. It is well known that skin temperature regulation is a complex system that depends on blood-flow rate, local structures of subcutaneous tissues and, mainly, the activity of the ANS (especially at the extremity sites). Anatomical and functional differences regarding left–right comparisons have been detected at several levels of neuroaxis [40, 41]. For example, an asymmetry has been shown in the descending pathways from the hypothalamus, and in the autonomic control of different organs [40], a degree of lateralization was found also in the anatomical projections from and to brain hemispheric areas associated with autonomic control [42, 43], and from the sympathetic premotor neurons to preganglionic segments [42]. For example, a study demonstrated that right arteries have significant higher innervation than left [44]. Therefore, a possible difference in left–right ANS activation could be explained by a hemispheric asymmetry in the response to dTMS due to a different cerebral hemispheric dominances. Obviously, these hypotheses need to be confirmed by targeted studies involving a larger population.

Several hypotheses can be raised about the mechanisms underlying the variation in the fingernail-bed temperature. The application of a linear regression model for investigating the effect of catecholamines on the left-hand fingernail-bed temperature changes showed a trend toward a significant positive effect of NE change on temperature variation in HF group but not in Sham. A comparable effect was not observed neither for EPI nor for the right hand.

Although in this study, a significant change in EPI and NE levels was not observed after a single session of HF dTMS compared to Sham, the evidence of a borderline significant impact of NE variation on fingernail-bed temperature change of left hand suggests that the mechanism by which dTMS can acutely affect body temperature in obesity may be related to an effect on SNS. This observation is indirectly supported by previous studies where HF repetitive TMS evoked a sympathetic activation measured with an increase in pupil diameter [45] and with an induced sympathetic skin response [46]. Conversely, application of low-frequency repetitive TMS to the PFC seems to affect SNS via a slight parasympathetic activation [47].

EPI and NE are both hormones and neurotransmitters; they are involved in several regulatory processes in the body by the brain. They are secreted into the bloodstream by the adrenal glands in response to stress, but they are also synthesized and released as neurotransmitters by axon terminals in the central nervous system and in sympathetic fibers of the ANS. Specifically, EPI is the main hormone secreted by the adrenal medulla and plays a key role in the responses to metabolic and global challenges to homeostasis, such as glucose deprivation, and in the response to emotional distress. For these reasons, EPI response is more closely linked to responses of the hypothalamic–pituitary–adrenocortical system than of the SNS. Norepinephrine is the main neurotransmitter of the SNS; it is responsible for tonic and reflexive in cardiovascular tone [48]. Norepinephrine preferentially stimulates α1- and α2-adrenoceptors, located on vascular smooth muscle cells, by eliciting vasoconstriction, and influencing blood flow, blood pressure, and consequently, body temperature in the extremities of the body [49–52]. Therefore, our hypothesis is that bilateral stimulation of both medial and lateral PFC, two brain areas which exert a well-defined control on ANS [53], could influence peripheral vasomotor activity through a modulatory impact on NE action. Furthermore, the lack of a significant impact of EPI on the temperature change of the fingernail-beds in both hands may be supportive evidence for the prevalent interconnection of NE with the SNS, and hence, for its prevalent role in thermoregulation.

Although a significant decrease of the fingernail-bed temperature of the right hand and to a lesser extent, of the left hand has been shown after a single session of HF dTMS, we found a positive correlation between fingernail-bed temperature of left hand and NE variations but no in fingernail-bed of the right hand. This result, which is a limitation of our study, could be explained considering that the human conduction system and the Kent bundles receive an appreciable sympathetic influence from the stellate ganglion (SG). Experimental studies found an asymmetric response to unilateral SG block and a dominance of the left SG [54, 55].

During a single treatment session, no significant variation in abdominal skin temperature was observed in HF arm compared to Sham. Being the temperature of the abdominal skin strongly conditioned by the subcutaneous adipose tissue acting as an insulating layer, we did not expect significant variations of temperature in this area after a single dTMS session. Furthermore, as previously reported, the main determinant of the skin temperature at the level of the limb extremities is the vascularization, mainly regulated by the SNS, but the abdominal adipose tissue is poorly vascularized and, probably, less sensitive to the potential dTMS-induced vasomotor effect.

Catecholamine-induced thermoregulation may result not only from the peripheral vasomotor activity by NE action on α-adrenoceptors, but also from the lipolytic effect of β-adrenoceptor agonism. Plasma increased catecholamine levels might increase resting energy expenditure, participating in maintaining body weight [48]. In this connection, BAT is the main responsible of non-shivering thermogenesis in humans and is deeply innervated by sympathetic fibers reaching their β3-receptors [56]. Although we were not able to acutely quantify the changes in intra-scapular and supra-clavicular skin temperature in the present study, it is conceivable that the SNS activation via dTMS might also induce an activation of BAT. However, this intriguing hypothesis needs to be tested especially in a longitudinal study in which the participants undergo repeated dTMS sessions.

Second achievement of our study is the establishment of a procedure (the combination of dTMS and IRT) which may be utilized beyond the treatment of pathophysiology of obesity, being extendable to all other conditions characterized by alteration of the ANS including cardiovascular diseases [57–59]. In fact, the correlation herein identified between heat production (measured with IRT) and NE blood level could be used as a physiological marker of SNS activity in several clinical and paraphysiological conditions including obesity and sport activity).

The main limitation of our study is that the temperature variations of fingernail-beds of the right hand and, to a lesser extent, of the left hand, and the blood level changes of EPI, NE, β-endorphin were assessed only acutely. Therefore, no data are currently available on the duration of dTMS-induced effects on body temperature variations in individuals with obesity, and assumptions about clinical implications of these findings should be proposed with caution. However, this study could constitute a proof of concept to be exploited by a longitudinal study, as previously done by us concerning the efficacy of dTMS on body weight [17].

In summary, this study suggests a potential effect of HF dTMS in modulating temperature in subjects with obesity, and sympathetic activity modulation represents one of the potential mechanisms via which dTMS exerts its thermoregulatory action.

Future longitudinal studies should be designed to analyze body temperature variations after repeated sessions of dTMS to confirm the potential role of dTMS in modulating ANS as well as BAT thermogenic activity.

## Data Availability

Individual participant data that underlie the results reported in this article, after de-identification (text, tables, figures, and appendices), will be available on https://zenodo.org/communities/multimedica/. Data will be available for investigators whose proposed use of the data has been approved by an independent review committee (learned intermediary) identified for this purpose and for individual participant data meta-analysis.

## Abbreviations

FFM: Fat-free mass
ANS: Autonomic nervous system
SNS: Sympathetic nervous system
NE: Norepinephrine
EPI: Epinephrine
BAT: Brown adipose tissue
WAT: White adipose tissue
NBS: Noninvasive brain stimulation
rTMS: Repetitive transcranial magnetic stimulation
dTMS: Deep transcranial magnetic stimulation
tDCS: Transcranial direct current stimulation
PFC: Prefrontal cortex
IRT: Infrared thermography
HF: High frequency
LF: Low frequency
ELISA: Enzyme-linked immunosorbent assay
EIA: Enzyme immunoassay
IQR: Interquartile range
BMI: Body mass index
SG: Stellate ganglion
